# High-resolution leak-out spectroscopy of HHe_2_^+^

**DOI:** 10.1039/d4cp04767b

**Published:** 2025-02-04

**Authors:** Thomas Salomon, Carlo Baddeliyanage, Carla Schladt, Irén Simkó, Attila G. Császár, Weslley G. D. P. Silva, Stephan Schlemmer, Oskar Asvany

**Affiliations:** a I. Physikalisches Institut, Universität zu Köln Zülpicher Str. 77 D-50937 Köln Germany asvany@ph1.uni-koeln.de; b New York University, Simons Center for Computational Physical Chemistry 24 Waverly Place, New York New York 10003 USA; c Institute of Chemistry, ELTE Eötvös Loránd University H-1117 Budapest Pázmány Péter sétány 1/A Hungary

## Abstract

Applying a novel and universal action spectroscopic technique, called leak-out spectroscopy, this paper revisits the *ν*_3_ proton shuttle motion of the symmetric linear molecule He–H^+^–He. For this, a 4 K cryogenic ion trap apparatus has been combined with a high-resolution quantum cascade laser operating around 1300 cm^−1^. Seven rovibrational lines of this fundamental three-nucleus-four-electron system are recorded, demonstrating the suitability of the leak-out method for such fundamental hydrogen–helium cations.

## Abstract 2

Applying a novel and universal action spectroscopic technique, called leak-out spectroscopy, this paper revisits the *ν*_3_ proton shuttle motion of the symmetric linear molecule He–H^+^–He.

## Graphical abstract

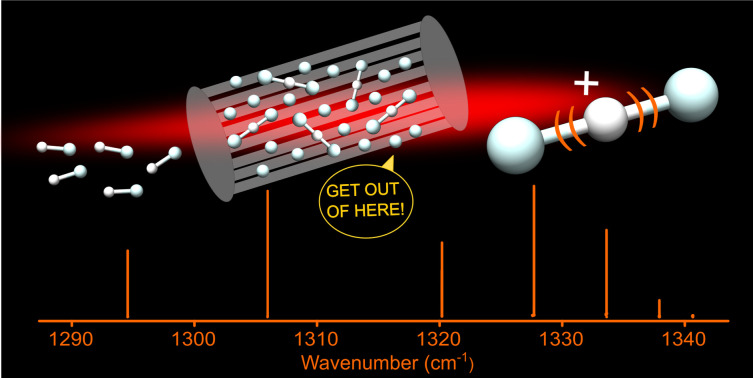

## 1. Introduction

A proton solvated by helium atoms, HHe_*n*_^+^, is an interesting molecular system. The first ion in this series, the H_2_-like and strongly bound HHe^+^ cation, has been known in the laboratory since 1925,^1^ has been investigated by high-resolution spectroscopy,^2–7^ and was detected in space quite recently.^8^ The next ion within this series, He–H^+^–He (ref. 9–16), is a linear three-nucleus-four-electron system. It is fairly strongly bound with a dissociation energy of *D*_0_ = 3931 ± 20 cm^−1^ (ref. 14), whereas all additional He atoms in higher order complexes (*n* = 3–6) are loosely bound to the central proton by less than 200 cm^−1^. The species *n* = 3–6 have been investigated by low resolution vibrational predissociation spectroscopy,^17^ in which the antisymmetric stretch (*ν*_3_) and bend (*ν*_2_) fundamentals of the He–H^+^–He core were observed around 1300 cm^−1^ and 850 cm^−1^, respectively. More recently, the *ν*_3_ mode of “naked” He–H^+^–He has been investigated in high resolution, with three rovibrational lines detected.^18^ This particular mode, *ν*_3_, is the proton shuttle motion and can be considered as a molecular realization of a Hertzian dipole, with its infrared (IR) intensity computed harmonically to be as large as 2661 km mol^−1^.^13^

The invention of the leak-out-spectroscopy method (LOS^19^) has boosted ion-trap-based spectroscopy of molecular ions, as it is a universal and close to background-free technique, with many recent applications to astrophysically relevant cations.^19–27^ In brief, LOS exploits the fact that the vibrational energy of a laser-excited ion can be converted into kinetic energy in a collision with a suitable neutral molecule or atom. These accelerated ions may then escape the ion trap and can be counted in a detector. By counting the “leaked-out” ions as a function of the laser wavelength, a spectrum is generated. In the current work, we revisit the *ν*_3_ mode of He–H^+^–He with the LOS method, using the very He atoms in the ion trap as the neutral collision partner required for LOS. The high-quality results obtained for HHe_2_^+^ encourage us to investigate further fundamental hydrogen–helium cations with LOS.

## 2. Experimental methods

The experiments of this study have been carried out in the cryogenic 22-pole ion trapping instrument COLTRAP.^28^ In brief, a pulse of several ten thousand HHe^+^ ions was generated in an ion source by electron impact ionization of an H_2_–He mixture, selected in a quadrupole mass spectrometer for a mass range 2–6 u (exact selection was considered not necessary), and then injected into the 22-pole trap.^29^ The trap was held at a temperature of *T* = 4 K and was constantly filled with He gas (∼10^15^ cm^−3^). During the trapping time of typically several 100 ms, the cold He gas fulfilled two roles: primarily, it enabled HHe_2_^+^ ions to be formed by 3-body collisions, and secondarily, it served as the collision partner required for the leak-out of the HHe_2_^+^ ions. For LOS, the ion ensemble was irradiated with narrow-bandwidth 7.5 μm IR radiation, which passed through the ion trap with a measured power on the order of 40 mW. The ions leaking out from the trap during the trapping time were selected in a second quadrupole for mass 9 u, and counted in a high-efficiency ion counter. The light source was a quantum cascade laser (Daylight Solutions) operating in the range of 1284–1355 cm^−1^, whose frequency was measured by a wavemeter (Bristol model 621 A-XIR).

## 3. Results and discussion

**Fig. 1:**
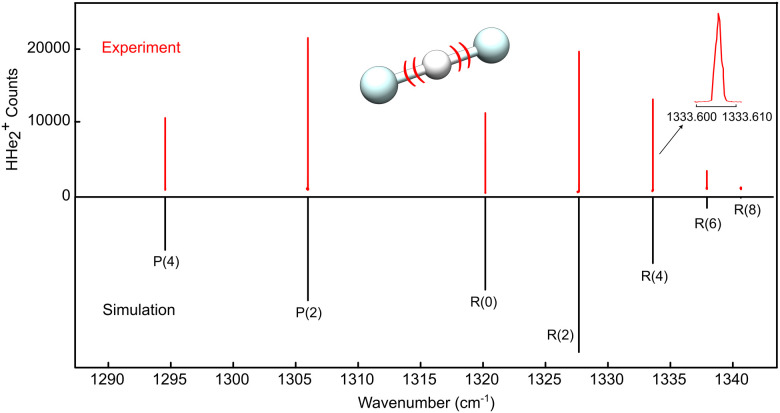
LOS spectrum of the *ν*_3_ antisymmetric stretching band of HHe_2_^+^. The upper part shows the experimental spectrum, obtained by individually targeting the transitions, as shown for the zoom of the line *R*(4). The lower part is a stick spectrum simulated at a temperature of 35 K using the fitted parameters from Table 2.

The high resolution LOS spectrum obtained for the proton-shuttle motion *ν*_3_ of HHe_2_^+^ is shown in Fig. 1. Similar to CO_2_, only levels with even rotational quantum number *J* are allowed in the ground state owing to nuclear spin statistics. An analysis of the observed line widths (∼60 MHz including an estimated laser linewidth of ∼30 MHz) yields a kinetic temperature of about *T*_kin_ ≈ 30 K, and the simulation of the intensity distribution seen in Fig. 1, obtained with PGOPHER,^30^ corresponds to a rotational temperature of *T*_rot_ = 35 K. While much lower temperatures could be obtained by optimizing some experimental settings (*e.g.* by better mass selection and storing less ions), the high temperature of the spectrum shown in Fig. 1 serendipitously permitted us to record as many as seven rovibrational lines, given in Table 1. The lines show excellent agreement, with a difference of only 0.26 cm^−1^, with accurate variationally calculated values (“first principles”, also listed in Table 1), obtained with the same methodology as used in ref. 16. This constant difference is solely due to the vibrational contribution, whereas the rotational structure in the ground and vibrationally excited states is matched extremely well. Performing a least-squares fit of the observed lines using a linear rotor Hamiltonian as implemented in PGOPHER, the spectroscopic parameters shown in Table 2 are obtained. As can be seen in Table 2, there is a large change of the rotational constant *B* upon vibrational excitation whose value decreases by about 10% in comparison to the ground state. This difference is caused by the shallow and anharmonic potential energy surface of HHe_2_^+^ that results in the asymmetric appearance of the *ν*_3_ band depicted in Fig. 1.

**Table 1:** Experimental and first-principles computed rovibrational transitions of HHe_2_^+^ (in cm^−1^)

	(*v*_3_,*J*′)	←	(*v*_3_,*J*′′)	Experiment	First principles
*P*(4)	(1,3)	←	(0,4)	1294.5488(4)	1294.28
*P*(2)	(1,1)	←	(0,2)	1305.9777(4)	1305.72
*R*(0)	(1,1)	←	(0,0)	1320.1882(4)	1319.93
*R*(2)	(1,3)	←	(0,2)	1327.6915(4)	1327.43
*R*(4)	(1,5)	←	(0,4)	1333.6069(4)	1333.35
*R*(6)	(1,7)	←	(0,6)	1337.9292(4)	1337.67
*R*(8)	(1,9)	←	(0,8)	1340.6515(4)	1340.39

**Table 2:** Spectroscopic parameters of HHe_2_^+^ (in cm^−1^), obtained by fitting the data given in Table 1 with the PGOPHER^30^ program, along with previously reported computational results^13,15,18^

	This work								
	Experimental		First principles		*Ab initio*, ref. 18		*Ab initio*, ref. 13		*Ab initio*, ref. 15
	*v*_3_ = 0	*v*_3_ = 1	*v*_3_ = 0	*v*_3_ = 1	*v*_3_ = 0	*v*_3_ = 1	*v*_3_ = 0	*v*_3_ = 1	*v*_3_ = 1
*ν*_3_		1315.8444(2)		1315.58		1306.2		1345.2	1318.6
*B*	2.36877(6)	2.17208(6)	2.3693	2.1726	2.3616	2.1491	2.3622	2.1506	
*D*	0.000056(2)	0.000052(2)	0.00005	0.00005	0.000046		0.00004		

## 4. Conclusion and outlook

In conclusion, the exceptional sensitivity of LOS and its application to the *ν*_3_ proton shuttle motion of HHe_2_^+^ allowed us to detect seven of its rovibrational lines, four of which are reported here for the first time. In comparison to our former work,^18^ a much better determination of the spectroscopic parameters of HHe_2_^+^ (see Table 2) could be achieved here. Our novel results will certainly facilitate astronomical searches for HHe_2_^+^, preferentially in the infrared region using the recently launched James Webb Space Telescope. After this successful demonstration, we plan to measure the bending mode *ν*_2_ or the combination band *ν*_1_ + *ν*_3_ of HHe_2_^+^, with calculated band origins of *ν*_2_ = 874.9 cm^−1^ and *ν*_1_ + *ν*_3_ = 2057.9 cm^−1^ (ref. 16), respectively. An equally attractive target is the fundamental H_2_ stretch in linear H_2_He^+^, or the D_2_ stretch in D_2_He^+^. The wavenumber of the latter is predicted to be at 1318 cm^−1^ (ref. 31), which we could confirm by recording its first rovibrational fingerprints, using the same quantum cascade laser as in this work.

## Data availability

All the important data (transition frequencies) are contained in Table 1.

## Conflicts of interest

There are no conflicts to declare.
